# Enhancing the Yield of a Lab-on-a-Disk-Based Single-Image Parasite Quantification Device

**DOI:** 10.3390/mi14112087

**Published:** 2023-11-11

**Authors:** Vyacheslav R. Misko, Ramadhani Juma Makasali, Matthieu Briet, Filip Legein, Bruno Levecke, Wim De Malsche

**Affiliations:** 1 µFlow Group, Department of Chemical Engineering, Vrije Universiteit Brussel, 1050 Brussels, Belgium; veaceslav.misco@vub.be (V.R.M.); ramadhani.makasali@sua.ac.tz (R.J.M.); matthieu.briet@vub.be (M.B.); filip.legein@vub.be (F.L.); 2 Department of Translational Physiology, Infectiology and Public Health, Ghent University, 9820 Merelbeke, Belgium; bruno.levecke@ugent.be; 3 Department of Bioengineering Sciences, Vrije Universiteit Brussel, 1050 Brussels, Belgium

**Keywords:** particle separation, parasite egg identification and quantification, diagnostic microfluidic device, extreme point of care

## Abstract

The recently proposed single-image parasite quantification (SIMPAQ) platform based on a Lab-on-a-Disc (LOD) device was previously successfully tested in field conditions, demonstrating its efficiency in soil-transmitted helminth (STH) egg detection and analysis on the level delivered by the current state-of-the-art methods. Furthermore, the SIMPAQ provides relatively quick diagnostics and requires small amounts of sample and materials. On the other hand, in a recent related study, it was revealed that the performance of the SIMPAQ method can be limited due to the action of the tangential Euler and Coriolis forces, and the interaction of the moving eggs with the walls of the LOD chamber. Here, we propose a new improved design that allows us to overcome these limitations and enhance the yield of the SIMPAQ LOD device, as demonstrated in experiments with a synthetic particle model system and real parasite eggs. Despite the simplicity, the proposed design modification is demonstrated to allow a substantial improvement in the yield of the SIMPAQ device, i.e., above 90% of parasite eggs and 98% of synthetic model particles were transported to the field of view. The new design proposed here will be further examined in the new generation of SIMPAQ devices within ongoing research on STH egg detection in field conditions.

## 1. Introduction

A Lab-on-a-Disc (LOD) device was recently developed [1] for the detection of soil-transmitted helminths (STHs), i.e., intestinal worms that infect humans and are spread through contaminated soil [2,3]. The LOD device is a centrifugal microfluidic platform based on centrifugation and flotation that isolates and collects eggs within an imaging zone using, e.g., saturated sodium chloride as the flotation solution. The main advantage of this device as compared to other diagnostic methods (see, e.g., [4,5,6,7,8]) is that it holds promise to provide quick diagnostics where needed (point-of-care (POC) testing), requiring small amounts of sample and materials only. It provides fast operation and an image of a packed monolayer of eggs collected within a single imaging zone or field of view (FOV). A distinct feature of this system is that a parasite egg monolayer can be formed by restricting the chamber height of the imaging zone to the size of a single egg (as low as 60 µm). The platform was successfully tested in Ethiopia, Tanzania, Uganda, and Kenya on infected human and animal samples for evaluation of the developed technology [9].

In turn, LODs are centrifugal microfluidic devices forming a subclass of integrated Lab-on-a-Chip (LOC) platforms [10,11,12,13] that have advantages such as portability, the use of small amounts of materials and reagents, faster reaction times and shorter time-to-result, and the programmability of process steps through varying the rotational conditions [11]. In addition to these, the LOD platform employs pseudo-forces generated during the rotation of the device: centrifugal force, the Coriolis force, and the angular-acceleration-generated Euler force [12]. Several applications including clinical chemistry, immunoassay, cell analysis [14,15], and nucleic acid tests [16,17,18] have been demonstrated on a spinning disc [19]. Some applications of these LOD platforms are sample-to-answer systems for biomedical POC and global diagnostics [20], liquid handling automation for the life sciences, process analytical techniques and cell line development for biopharma, and monitoring of the environment, infrastructure, industrial processes, and agrifood [21,22]. The LOD platform has been applied for the detection and molecular analysis of pathogens [23] such as, e.g., *Salmonella*, a major food-borne pathogen [24,25]. Recently, the possibilities of employing LOD platforms for the detection of COVID-19 have been discussed in the literature as well [26]. 

The effect of the lateral walls of a LOD device on the dynamics of a model system of particles with a density lower than that of the solvent (modelling parasite eggs) has been analyzed theoretically and experimentally [27]. It has been shown that the trajectory of a particle moving under the action of the centrifugal force is deflected in the tangential direction by the inertial Coriolis and Euler forces, and in this situation, the particle easily reaches the lateral walls of the narrow channel. 

**Figure 1:**
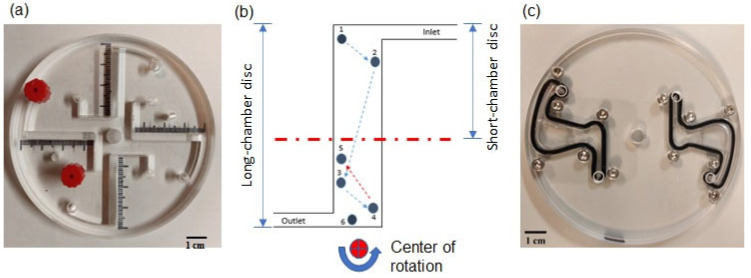
A long-chamber disc [27] (**a**). A typical trajectory of a particle (experiment) showing zig-zag and backwards motion near the center of rotation in a long-chamber disc [27]; the truncation of the chamber in a short-chamber disc is schematically shown by the dash-dotted red line (**b**). Short-chamber disc (**c**).

A particle trajectory is affected by the Coriolis force, $-2m\vec{\omega }\times \vec{v}$, and the Euler force, $-m\frac{d\vec{\omega }}{dt}\times \vec{r}$. These forces, which are tangential to either the velocity vector of the moving particle (the Coriolis force) or the radial direction (the Euler force), lead to a deviation in the direction of motion of particles (eggs) from the direction of the centrifugal force, $-m\vec{\omega }\times \left(\vec{\omega }\times \vec{r}\right)$ (i.e., the radial direction) [27]. Being confined in a narrow channel, a particle (egg) can therefore easily reach the lateral walls of the channel. The walls, depending on the angle they form with the radial direction, can guide the particle either in the same or in the opposite direction to the centrifugal force, thus resulting in unusual particle trajectories including zig-zag or backwards particle motion [27] (see Figure 1b). This effect is pronounced in the case of short operation times and for repeated ramping up and down cycles when the acceleration of the angular rotation, and, thus, the Euler force, is considerable. This behavior has been observed in experiments with particles of density lower than that of the solvent, i.e., modeling eggs. 

The revealed unusual motion patterns represent undesirable effects that can prevent some parasite eggs from reaching the FOV of the LOD device, thus limiting its efficiency. Since the density of parasite eggs is lower than that of the solvent, they move towards the center of rotation during centrifugation. It is worth noting that the revealed behavior occurs mainly near the center of rotation where the centrifugal force is small, and the interplay of the tangential Euler and Coriolis forces and the interaction with the walls could cause the backward motion [27]. Therefore, the undesirable effects occur near the entrance to the FOV, as confirmed by the experimental observations. In addition, the channel has a smooth step near the entrance to the FOV [18,27], which can trap some eggs before they reach the FOV considering the strong decrease in the centrifugal force near the entrance to the FOV, as compared to its values in the rest of the channel.

One way to overcome this undesired effect is to substantially increase the rotation speed of the disc, which leads to very large tangential forces exerted on the eggs at the disc periphery that could be even damaging to the eggs (due to collision with the lateral walls). In addition, an excessive rotation speed increases the chance of a sudden leakage or disc precession. Thus, increasing the rotation speed is not an optimal solution. Alternatively, we propose to *reduce* the length of the channel (Figure 1) and, in this way, decrease the difference between the inertial forces acting on the eggs at the disc periphery and at the entrance to the FOV. In other words, we simply remove a part of the channel where the undesirable effects, i.e., zig-zag and backwards motion, can occur. In this way, we expect that eggs will reach the FOV at a reasonable operational rotation speed, enhancing the yield of the LOD device. In this work, we performed experiments with synthetic particles modeling eggs and with real parasite eggs in a new LOD device with an improved design, and we compare the obtained results with those for the original (“long”) disc [18,27]. 

## 2. Experimental Setup and Materials

### 2.1. Imaging Setup

**Figure 2:**
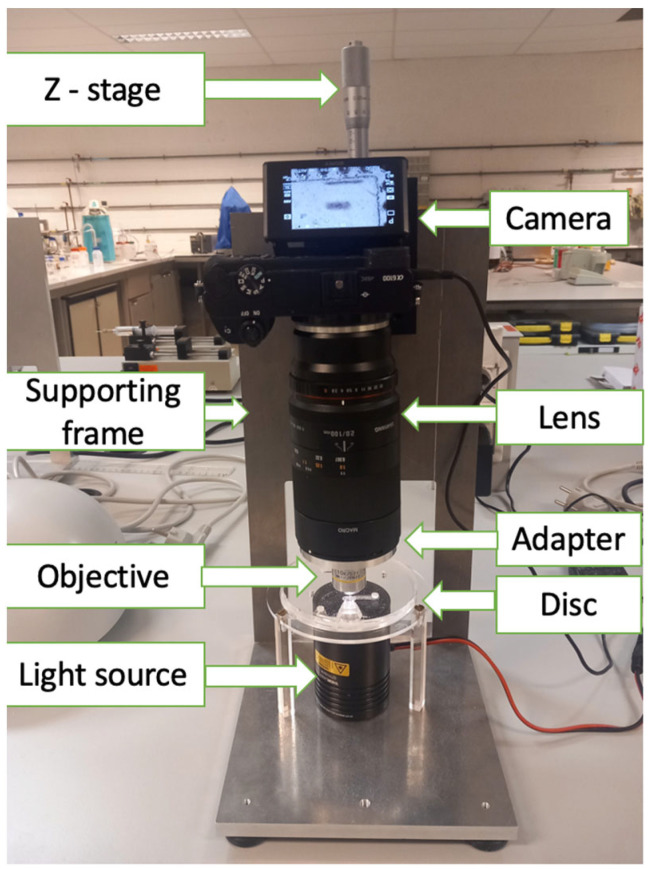
The imaging setup.

The imaging setup is shown in Figure 2. For the capture and storage of a single image for future examination, a Sony α6100 camera ( Sony Group Corporation, Minato, Tokyo, Japan) was used throughout the experiments. The camera was connected to a Samyang Macro lens 2.8/100 mm (Samyang Optics, Masan, South Korea). With the aid of an adaptor, the Macro lens was connected to an objective lens of 10× or 20× magnification power, depending on the experiments. To increase the visibility of the artificial parasite eggs (polystyrene particles) and the actual STH eggs in the LOD, the imaging setup was connected to a halogen light source (Quartz Tungsten-Halogen lamp, Thorlabs Inc., Newton, NJ, USA).

### 2.2. LOD Device: Short-Chamber vs. Long-Chamber Disc

**Figure 3:**
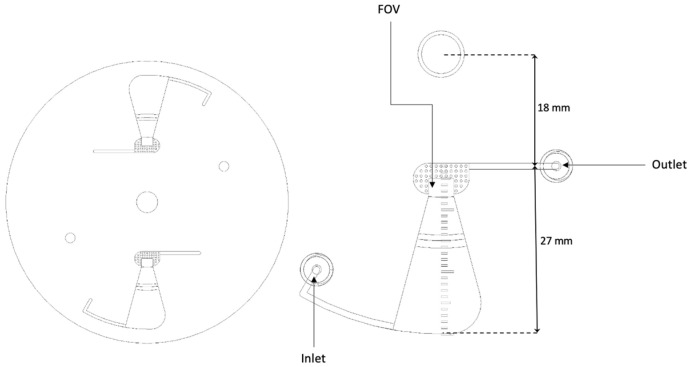
Computer-aided design (CAD) image of the short-chamber LOD with indications of the total length of the chamber and the length from the chamber to the center of rotation.

**Figure 4:**
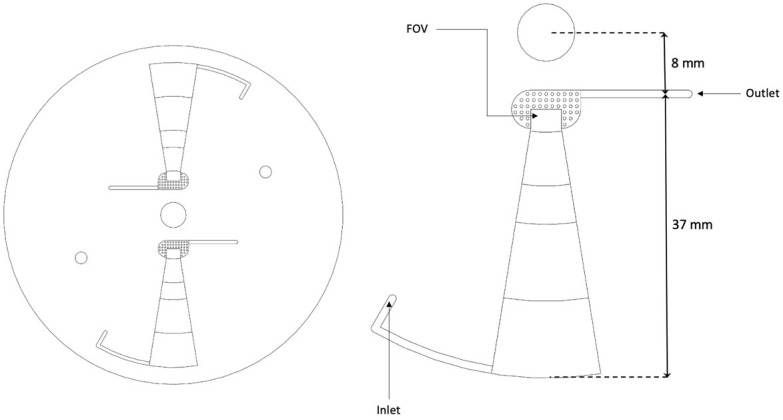
CAD image of the long-chamber LOD.

The newly designed short-chamber LOD (Figure 3) produced from Poly (Methyl Methacrylate) (PMMA) plastic possesses a shorter convergence chamber of 27 mm in length with a width and depth that decrease in size from the periphery toward the rectangular section (collection zone/FOV). The chamber has a curved corner facing the sample entrance channel, and the length from the chamber to the center of the disc is 18 mm. The long-chamber LOD (Figure 4) was also produced from PMMA and fabricated in such a way that it possesses a longer convergence chamber of 37 mm in length with a width and depth that decrease from the periphery towards the rectangular section (collection zone/FOV). Also, the chamber has an angled corner opposite the sample entrance channel. The distance from the chamber to the center of the disc is 8 mm.

**Figure 5:**
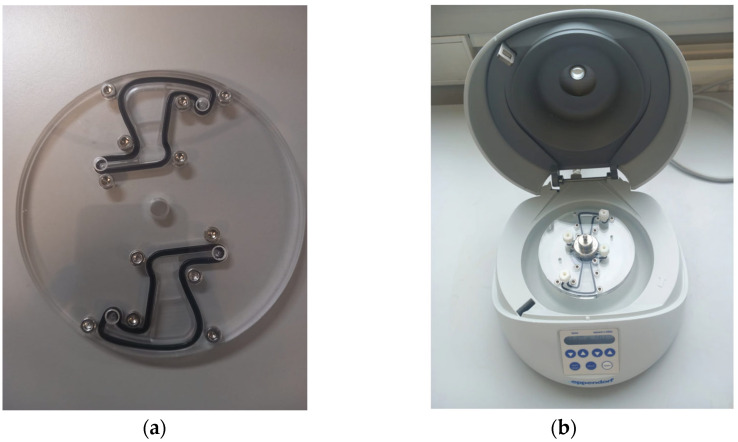
(**a**) A photograph of the modified reversible bound short-chamber LOD device, and (**b**) a customized mini centrifuge containing a reversibly bonded long-chamber LOD device.

Figure 5 presents photographs of the modified reversible bound short-chamber LOD device and a customized mini centrifuge (Eppendorf^®^ Centrifuge MiniSpin G; Eppendorf, Hamburg, Germany) [18,27] containing a reversibly bonded long-chamber LOD device.

### 2.3. Flotation Solution for Particles and Eggs

To prepare the flotation solution, 300 g of NaCl salt was measured on a weighing scale and dissolved in 1 L of distilled water (Millipore Synergy UV, Spectralab Scientific lnc., Markham, ON, Canada). A magnetic stirrer together with a magnet rod was used to hasten the dissolution of the mixture until the NaCl salt was completely dissolved. Then, the density of the flotation solution was measured using a hydrometer and found to be 1.175 g/mL. The density of the flotation solution of 1.175 gmL^−1^ is lower than the densities of both the red polystyrene (PS) particles and STH eggs. The density of the red PS particles is 1.05 gmL^−1^, the density of *Ascaris* eggs is 1.11 g/mL, the density of hookworm eggs is 1.055 g/mL, and the density of *Trichuris* eggs, which are the heaviest among the STH eggs, is 1.15 g/mL [28].

Experiments were conducted with samples containing Ascaris eggs stored in ethanol, which can affect their density and migration behavior in the disk. The suspension also contained other particles and could also contain other parasites. In a follow-up study, we will conduct experiments directly after the collection of stool samples (avoiding storage in methanol) and will conduct a more detailed analysis on the individual egg level to explore potential differentiation of the parasite species that are present.

## 3. Results and Discussion

### 3.1. The Influence of Chamber Design on the Trajectories of Polystyrene Particles

The experiment was conducted by initially introducing a single red PS particle into both the short-chamber LOD device and the long-chamber LOD device, and centrifugation was performed at rotation speeds of 800 rpm (providing acceleration from 15*g*, near the FOV, to 32*g*, at the periphery, where *g* = 9.81 m/s^2^ is the acceleration due to gravity at the Earth’s surface), 1000 rpm (23*g* to 50*g*), 1500 (53*g* to 113*g*), and 2000 rpm (94*g* to 201*g*) in a 5 s repeated centrifugation cycle until the PS particle reached the FOV. The positions of the particles were recorded after each centrifugation cycle. (We note that the trajectories of the moving particles were not tracked in a continuous manner. We only recorded the positions of the particles after each rotation cycle.) For each rotation speed, the experiment was repeated five times.

**Figure 6:**
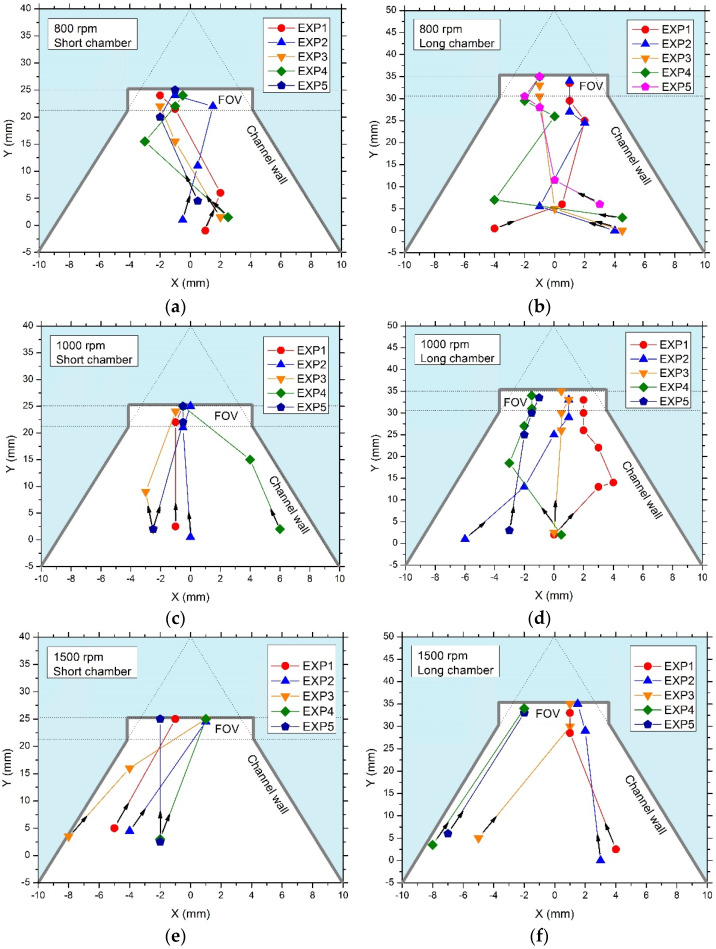
Trajectories of the red polystyrene particles in the short-chamber LOD and long-chamber LOD. (**a**,**c**,**e**): trajectories of five red polystyrene particles in short-chamber LODs. (**b**,**d**,**f**): trajectories of five red polystyrene particles in the long-chamber LODs. (**a**,**b**): rotation speed of 800 rpm, (**c**,**d**): rotation speed of 1000 rpm, and (**e**,**f**): rotation speed of 1500 rpm. For each rotation speed and each LOD device, there were repeated cycles of 5 s centrifugation.

At a rotation speed of 800 rpm, two observations were made: (i) First, the time needed for PS particles to reach the FOV was appreciably longer than that for a higher rotation speed. Thus, in both short-chamber and long-chamber LODs, PS particles had multiple *x*- and *y*-coordinate positions (see Figure 6a,b) before they reached the FOV. In the short-chamber LOD device, only 40% of PS particles (EXP 3 and EXP 5) reached the FOV within 10 s, while the remaining 60% of the particles (EXP 1, EXP 2, and EXP 4) took 15 s to migrate to the FOV (Figure 5a); in the long-chamber LOD device, all PS particles, 100% (EXP 1, EXP 2, EXP 3, EXP 4 and EXP 5), took 20 s to migrate to the FOV (Figure 6b). (ii) Second, for a rotation speed of 800 rpm in both LOD devices, the trajectories of the PS particles showed several zig-zag patterns (which were even more pronounced in rectangular chambers where backwards motions could also be observed, see Figure 1b) while migrating to the FOV (Figure 6a,b). These two observations resulted from the application of a relatively low rotation speed (800 rpm) that caused the generation of a small relative centrifugal force (RCF), as discussed in [29]. Therefore, the generated small relative centrifugal force caused the denser flotation solution to move slowly to the periphery of the chamber and led to the generation of a small buoyant force that caused the PS particles to move slowly to the FOV. During the motion, the inertial pseudo-forces, i.e., the Euler and Coriolis forces, caused the sideways motion of the PS particles, resulting in multiple zig-zag parts in the trajectories observed in both LOD devices. Similar trajectories with zig-zag parts of moving PS particles in the LOD device were observed in a recent study [27], where it was revealed that the magnitude of the rotation speed is one of the factors that can influence the direction of motion of a particle in a rotating disk, when the interplay of the Euler and Coriolis forces with the interaction of the particle with the chamber walls can cause the particle to move either away or towards the center of rotation [27].

The relation between the magnitude of the rotation speed and the occurrence of zig-zag parts in the trajectories of the PS particles was also proven in the experiments when the rotation speed was increased. Thus, at a rotation speed of 1000 rpm, zig-zag parts in the trajectories of the PS particles were much more pronounced in the long-chamber LOD device than in the short-chamber LOD device (see Figure 6c,d). This observation was also supported in [29], where the authors mentioned that the relative centrifugal force also depends on the distance of the particles from the center of rotation. Therefore, this could be the reason why there were fewer zig-zag parts in the trajectories of PS particles in the short-chamber LOD device, i.e., because the chamber is far from the center of rotation, and, therefore, the generated relative centrifugal force was somehow high enough to generate the buoyant force that drove the PS particles to the FOV with fewer zig-zag patterns in their trajectories.

At a rotation speed of 1500 rpm, 80% of the PS particles (EXP 1, EXP 2, EXP 4, and EXP 5) in the short-chamber LOD device (Figure 6e) reached the FOV without undergoing zig-zag parts in their trajectories, whereas with the same rotation speed, in the long-chamber LOD device, only 40% (EXP 1 and EXP 2) managed to reach the FOV without undergoing zig-zag trajectories (Figure 6f). This result indicates that in the long-chamber LOD device, the Euler and Coriolis forces exerted on the particles are strong enough to cause the zig-zag patterns in the trajectories of the moving PS particles even when the rotation speed is 1500 rpm (see Figure 6e,f).

Finally, at a rotation speed of 2000 rpm, in both LOD devices, 100% of the PS particles managed to reach the FOV without undergoing zig-zag patterns in their trajectories. In addition, the time taken for the PS particles to reach the FOV was 5 s for all the particles in both LOD devices. Figure S1 in the Supporting Information presents images of multiple red polystyrene particles collected in the FOV of the LOD device.

The results of this experiment indicate that (i) there is a significant relation between the magnitude of the rotation speed and the trajectories of the PS particles. It was observed in the experiment that zig-zag parts in the trajectories of the particles decreased with increasing rotation speed. Additionally, (ii) this undesired effect is essentially suppressed in the short-chamber LOD, as compared to the long-chamber LOD.

### 3.2. Efficiency of the Short-Chamber LOD Device vs. the Long-Chamber LOD Device in Delivering Polystyrene Particles to the FOV

To carry out this experiment, 100 red polystyrene particles were introduced into both the short-chamber LOD device and the long-chamber LOD device, and centrifugation was performed at rotation speeds of 1000 rpm, 1500 rpm, and 2000 rpm for 1 min per rotation speed. The experiment was repeated three times at each rotation speed.

The result of this experiment showed that, at the rotation speed of 1000 rpm, 92 ± 2.7% of the PS particles were delivered to the FOV in the short-chamber LOD device, while the long-chambered LOD device showed a lower efficiency in delivering PS particles to the FOV (82.7 ± 3.2%). At a rotation speed of 1500 rpm, the fraction of PS particles that reached the FOV in the short-chamber LOD device increased to 95 ± 2.0%, while for the long-chamber LOD device, this fraction was 91 ± 3.0% (Table 1). At a rotation speed of 2000 rpm, the fraction of PS particles delivered to the FOV in the short-chambered LOD device reached the value of 98.7 ± 2.3%, where in two out of three experiments, the short-chamber LOD delivered 100% of the PS particles to the FOV. For the long-chamber LOD, the corresponding fraction of PS particles delivered in the FOV appeared to be 93.33 ± 1.5% in three repeated experiments. These results indicate that the number of PS particles in the FOV increased with an increase in rotation speed in both LOD devices, and that the fraction of particles delivered to the FOV was higher for the short-chamber LOD device. 

So far, 2000 rpm was the rotation speed with the highest yield in both the short-chamber and long-chamber LOD devices. However, the short-chamber LOD device was superior to the long-chamber LOD, since it allowed the delivery of 98.67 ± 2.31% of the PS particles to the FOV. 

**Figure 7:**
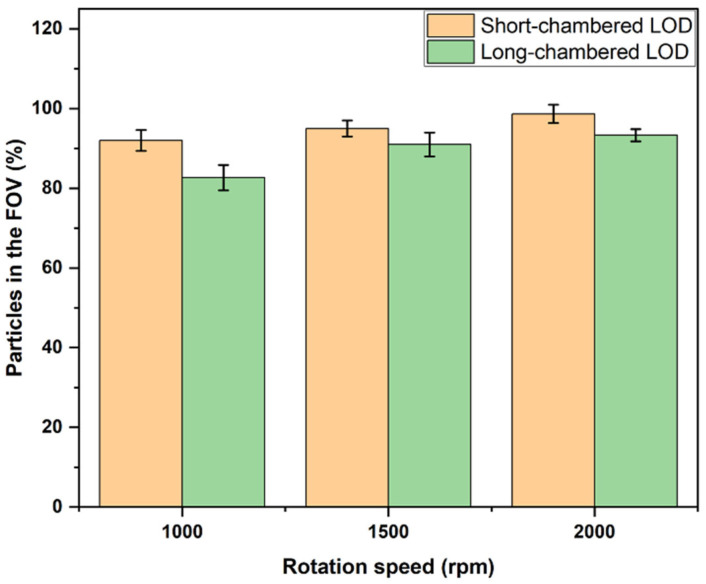
The average fractions of PS particles in the FOV versus rotation speeds in short-chamber and long-chamber LODs.

In the long-chamber LOD device, generally, the PS particles that were not able to reach the FOV were observed near the entrance of the FOV, which is indicative of the inability of the rotation speeds used in this experiment (1000 rpm, 1500 rpm, and 2000 rpm) to generate sufficient force for about 5% of PS particles to reach the FOV. This observation is in line with the conclusions of [30], claiming that the buoyant force increases when a cargo moves to pass a large depth. Therefore, when moving from the periphery of the chamber towards the center of rotation of the LOD, the buoyant force that drives PS particles to reach the FOV decreases, and a higher rotation speed is needed to generate more force to compensate this decrease in the buoyant force. Furthermore, the many-particle effects, such as their aggregation and interaction with the boundaries, lead to a further decrease in the driving force, requiring an even higher rotation speed to deliver PS particles to the FOV. In case of a single particle, the situation is different: at a rotation speed of 2000 rpm, the long-chamber LOD delivered a single red polystyrene particle to the FOV in a very short time. However, in the case of the short-chamber LOD device, the situation of many PS particles moving together to the FOV was not an issue because, as the results at the rotation speed of 2000 rpm show, the short-chamber LOD device delivered 100% of the PS particles to the FOV in two out of three experiments. This indicates that, since in the short chamber, both the initial and final points of the trajectory of moving PS particles are far from the center of rotation of the LOD, the rotation speed applied in this experiment generated sufficient buoyant force to deliver almost all the red polystyrene particles (>95%) to the FOV. The results for the short-chamber and long-chamber discs and for the rotation speeds of 1000, 1500, and 2000 rpm are summarized in Figure 7. 

Therefore, these experiments demonstrated that the efficiency of the newly designed short-chamber LOD in delivering PS particles to the FOV is essentially higher than the corresponding efficiency of the long-chamber LOD device of the previous generation. Already at a relatively moderate rotation speed of 2000 rpm, which is also safe in terms of, e.g., potential leakage or precession (which can be observed at extremely high rotation speeds), the new design, i.e., the short-chamber LOD, provides excellent efficiency in delivering PS particles to the FOV.

### 3.3. Efficiency of Short-Chamber and Long-Chamber LODs in Delivering STH Eggs to the FOV

This experiment was conducted by initially taking 100 μL of the stock sample containing purified eggs and mixing it with 100 μL of distilled water. The mixture was centrifuged at a rotation speed of 1500 rpm for 5 min. Following the centrifugation, the supernatant was discarded, and the sediments were resuspended by adding 500 μL of the flotation solution of 1.175 g/mL density containing 0.05% Tween-20. Then, 500 μL of the suspension solution was loaded into the chambers of the short-chamber LOD device and the long-chamber LOD device, which were prefilled with the flotation solution of 1.175 g/mL density containing 0.05% Tween-20, and both devices were centrifuged at rotation speeds of 1000 rpm (23*g* to 50*g*), 2000 rpm (corresponding to the acceleration of 94*g* to 201*g*), 3000 rpm (211*g* to 453*g*), and 4000 rpm (376*g* to 805*g*) for 1 min at each rotation speed. The experiment was repeated four times for each rotation speed and LOD device.

**Figure 8:**
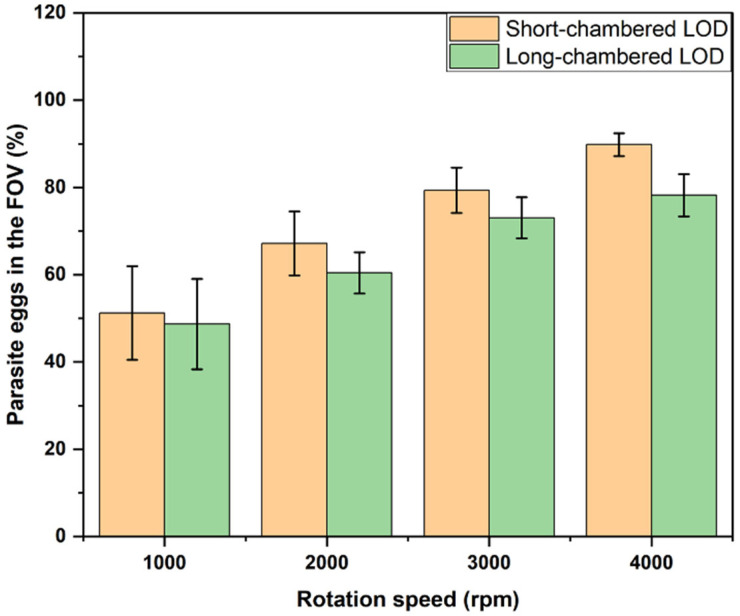
The average fractions of STH eggs in the FOV against rotation speeds in the short-chamber LOD and the long-chamber LOD.

The results of this experiment are, in general, in good agreement with those described above for PS particles, indicating that the model system fairly reproduced the properties of real parasite eggs. As in the case of PS particles, the higher the rotation speed, the higher the fraction of the eggs delivered to the FOV. Also, the short-chamber LOD device showed a higher efficiency in delivering eggs to the FOV as compared to the long-chamber LOD. The results for the short-chamber LOD vs. the long-chamber LOD, for different rotation speeds of 1000 rpm, 2000 rpm, 3000 rpm, and 4000 rpm, are summarized in Table 2. Since we used the same flotation solution (density: 1.175 g/mL) for both systems—PS particles (density: 1.05 g/mL) and STH eggs (density: 1.15 g/mL)—the density contrast for STH eggs was lower than that for PS particles, requiring a higher rotation speed to achieve the same output, i.e., the fraction of the eggs delivered to the FOV. Therefore, the rotation speed was increased to 4000 rpm in the experiments with STH eggs. The fractions of STH eggs delivered to the FOV for the short-chamber and long-chamber LODs, for the rotation speeds of 2000 rpm, 3000 rpm, and 4000 rpm, are shown in Figure 8 as the average (over four different experiments) fractions of STH eggs delivered to the FOV for the short-chamber and long-chamber LODs. As shown in Figure 8, the fraction of STH eggs delivered to the FOV gradually increased with the rotation speed for both the LOD devices, from about 50% at 1000 rpm to about 90% for the short-chamber LOD and about 80% for the long-chamber LOD at 4000 rpm. For all the rotation speed values, the efficiency in delivering STH eggs to the FOV for the short-chamber LOD was superior to that for the long-chamber LOD device.

**Figure 9:**
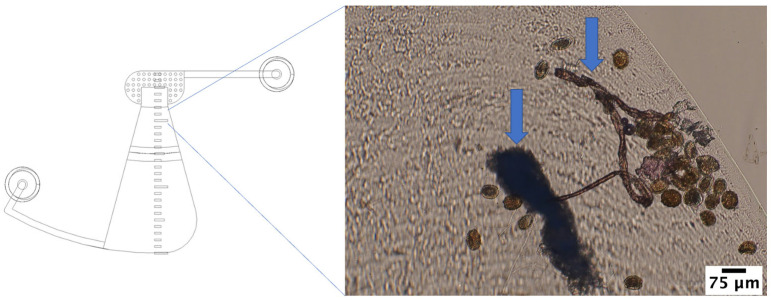
Some STH eggs got stuck on the way to the FOV due to fecal debris. Arrows indicate fecal debris.

During this experiment, some of the STH eggs were blocked by fecal debris that had a low density comparable to the density of the STH eggs (see Figure 9) while migrating to the FOV in both the short-chamber LOD device and the long-chamber LOD device. Therefore, secondary purification of the stock sample to obtain a clear sample with STH eggs without much fecal debris was performed, as well as an experiment to investigate the efficiency of the short-chamber LOD device and the long-chamber LOD in delivering the real STH eggs to the FOV.

Further, Figures S1 and S2 in the Supporting Information present images of multiple STH eggs collected in the FOV of the LOD device.

## 4. Conclusions

The promise of the microfluidic Lab-on-a-Disc (LOD) device for the quantification and identification of STH eggs to diagnose STH infections was recently shown. However, the developed LOD device with chambers extending from the edge of the disk to the close vicinity of the center of rotation (“long-chamber” LOD device) is confronted with a loss of some STH eggs from the FOV. Therefore, in this work, a new design for the disk with truncated chambers that end further away from the center of rotation (“short-chamber” LOD device) was developed, and its efficiency was compared with that of the long-chambered LOD device.

To achieve this goal, the influence of the chamber design on the trajectories of red polystyrene (PS) particles (which served as a model system for STH eggs) to the FOV was investigated. The zig-zag parts in the trajectories of the PS particles, which always occurred at lower rotation speeds, were eliminated in both the long-chamber LOD device and the short-chamber LOD device at 2000 rpm rotation speed. However, at a rotation speed of 1500 rpm, the short-chamber LOD device was able to deliver 80% (four out of five) of the PS particles to the FOV without zig-zag trajectory patterns, whereas the long-chamber LOD device was able to deliver only 40% (two out of five) of the PS particles to the FOV. This result indicates that even at a 1500 rpm rotation speed, the newly designed short-chamber LOD device can generate enough relative centrifugal force to overcome, to a large extent, the effects of the Euler force and Coriolis force that cause undesired distortions (like zig-zagging and backward motion) of the trajectories of the PS particles.

The efficiency of the short-chamber LOD device and long-chamber LOD device in delivering PS particles to the FOV was investigated. The results revealed that at the rotation speed of 1000 rpm, the short-chamber LOD device delivered 92 ± 2.7% of the PS particles to the FOV, whereas the long-chamber LOD device delivered 82.67 ± 3.2% of the PS particles to the FOV. At the rotation speed of 1500 rpm, the short-chamber LOD device delivered 95 ± 2% of the PS particles to the FOV, whereas the long-chamber LOD device delivered 91 ± 3%. At the rotation speed of 2000 rpm, the short-chamber and long-chamber LOD devices delivered, correspondingly, 98.67 ± 2.3% and 93.33 ± 1.5% of the PS particles to the FOV. In both the short-chamber and long-chamber LOD devices, the number of PS particles delivered increased with an increase in rotation speed. However, at each rotation speed used, the short-chamber LOD device delivered more PS particles to the FOV than the long-chamber LOD device. This is related to the fact that when placed further away from the center of rotation (in the short-chamber LOD device), the FOV becomes more easily reachable by moving particles that are driven by a stronger centrifugal force than in case of a long-chamber LOD device, where the FOV is placed in the vicinity of the center of rotation and the centrifugal force becomes minimal even at high rotation speed. 

We further investigated the efficiency of the short-chamber LOD device and long-chamber LOD device in delivering STH eggs to the FOV. The result obtained in this investigation is consistent with the result obtained when PS particles were used. Thus, the number of STH eggs in the FOV increased with increasing rotation speed. Like in the case of the PS particles, at each rotation speed used, the short-chamber LOD device delivered more STH eggs to the FOV than did the long-chamber LOD device. Therefore, the enhanced efficiency of the newly designed short-chamber LOD device in delivering STH eggs to the FOV for analysis was demonstrated.

The sample preparation step using a 200 μm filter membrane and a 20 μm filter membrane improved the yield of STH eggs in the FOV of the LOD devices. However, fecal debris that managed to pass the 200 μm filter membrane was still observed to be an obstacle to some STH eggs in reaching the FOV. Based on the results obtained in this work, it was found that the short-chamber LOD device overcame, to a large extent, the problem of some STH eggs not reaching the FOV. Furthermore, to increase the sensitivity and to make the short-chamber LOD device more user-friendly in field conditions, a 200 μm filter membrane and a 20 μm filter membrane could be incorporated into the chambers of the short-chamber LOD device so that the sample preparation step and the separation procedure could be integrated at the same time in the LOD technique. The new design analyzed in this work (as well as the further modifications proposed above) will be further examined in the new generation of SIMPAQ devices within ongoing research on STH egg detection in field conditions.

## Figures and Tables

**Table 1:** Proportion of red polystyrene particles in the FOV of the short-chamber LOD device and long-chamber LOD device, for rotation speeds of 1000 rpm, 1500 rpm, and 2000 rpm.

LOD	Red Polystyrene Particles in the FOV (%)		
	1000 rpm	1500 rpm	2000 rpm
Short-chamber LOD device	92 ± 2.7%	95 ± 2.0%	98.67 ± 2.3%
Long-chamber LOD device	82.67 ± 3.2%	91 ± 3.0%	93.33 ± 1.5%

**Table 2:** Proportion of STH eggs in the FOV of the short-chamber LOD device and the long-chamber LOD device, for rotation speeds of 1000 rpm, 2000 rpm, 3000 rpm, and 4000 rpm.

LOD	STH Eggs in the FOV (%)			
	1000 rpm	2000 rpm	3000 rpm	4000 rpm
Short-chamber LOD device	51.23 ± 10.7%	67.17 ± 7.3%	79.35 ± 5.2%	89.82 ± 2.6%
Long-chamber LOD device	48.7 ± 10.3%	60.43 ± 4.7%	73.03 ± 4.7%	78.21 ± 4.9%

## Data Availability

Data are contained within the article and Supplementary Materials.

## Supplementary Materials

The following supporting information can be downloaded at: https://www.mdpi.com/article/10.3390/mi14112087/s1, Figure S1. Polystyrene red particles in the FOV. The images were captured with an objective magnification of 20×. Figure S2. STH eggs in the image captured under objective magnification of 20×.
