# Supplementary material for: Enhancing the Yield of a Lab-on-a-Disk-Based Single-Image Parasite Quantification Device
Source: Micromachines (Basel). 2023 Nov 11;14(11):2087. doi: 10.3390/mi14112087 (PMC10672913; doi:10.3390/mi14112087)
Supplement: Supplementary file 1 [file micromachines-14-02087-s001.zip › micromachines-2663952-supplementary.pdf]

micromachines

MDPI

Supporting Information

Enhancing the Yield of a Lab-on-a-Disk-Based Single-Image Parasite Quantification Device

Vyacheslav R. Misko [1], Ramadhani Juma Makasali [1], Matthieu Briet [1], Filip Legein [1], Bruno Levecke [2] and Wim De Malsche [1,3,*]

[1] µFlow Group, Department of Chemical Engineering, Vrije Universiteit Brussel, 1050 Brussels, Belgium; veaceslav.misco@vub.be (V.R.M.); ramadhani.makasali@sua.ac.tz (R.J.M.); matthieu.briet@vub.be (M.B.); filip.legein@vub.be (F.L.)
[2] Department of Translational Physiology, Infectiology and Public Health, Ghent University, 9820 Merelbeke, Belgium; bruno.levecke@ugent.be
[3] Department of Bioengineering Sciences, Vrije Universiteit Brussel, 1050 Brussels, Belgium
* Correspondence: wim.de.malsche@vub.be; Tel.: +32-2-6293781

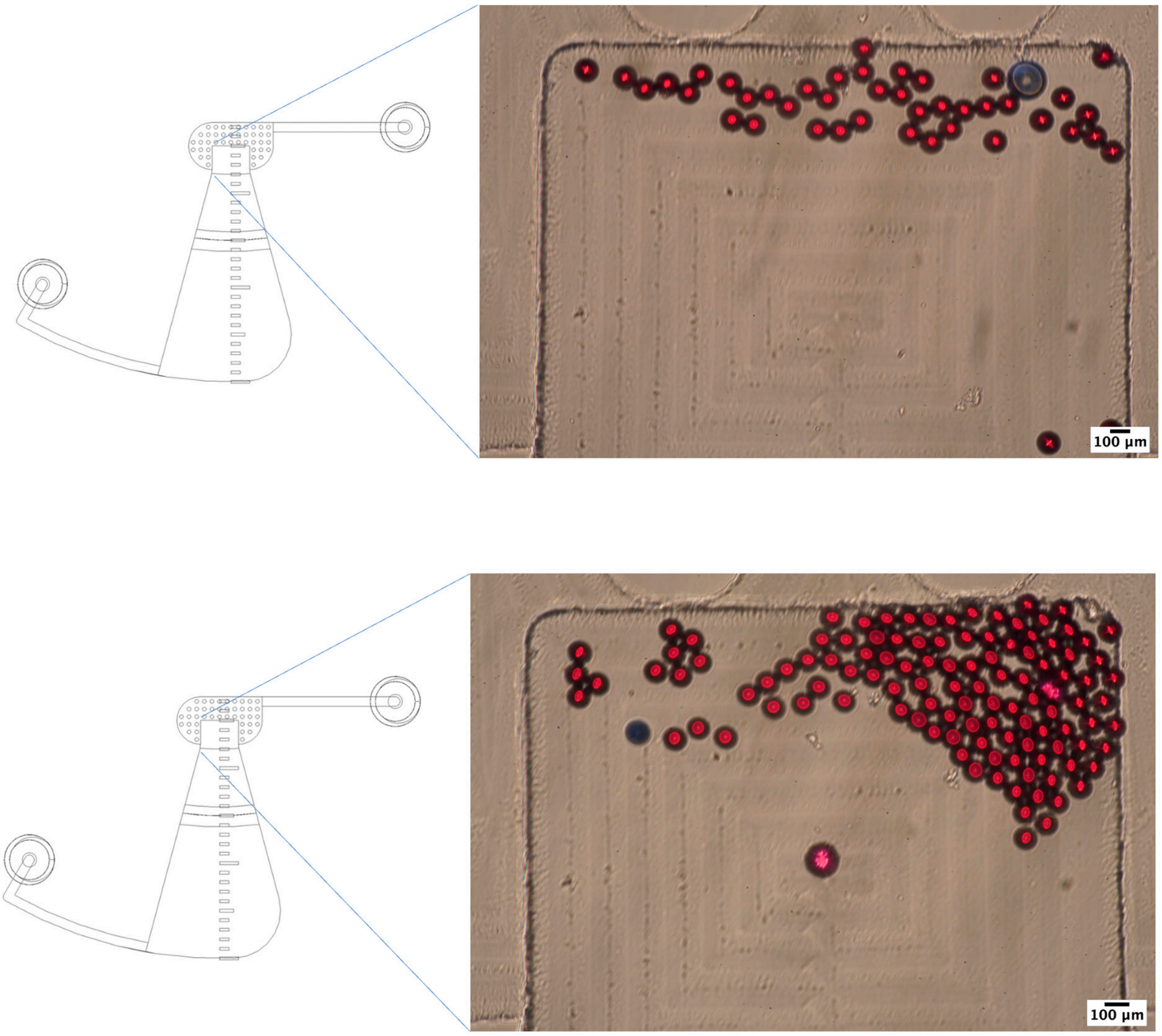

Figure S1. Red polystyrene particles in the FOV. The images were captured under 20× objective magnification.

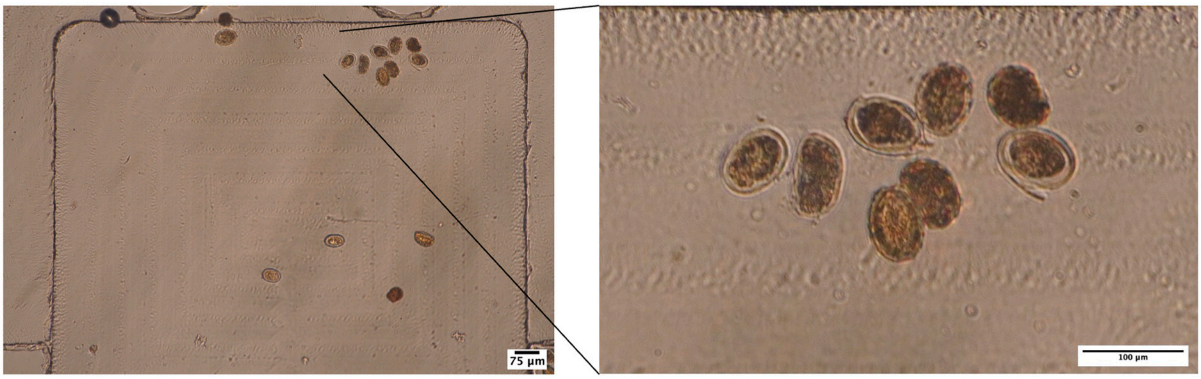

Figure S2. STH eggs in images captured under 20× objective magnification.
